# Exploring Potential Orbital Metastatic Pathways in Sinonasal Mucosal Melanoma: A Case Report

**DOI:** 10.1155/crot/6637565

**Published:** 2024-11-29

**Authors:** W. F. Julius Scheurleer, W. Weibel Braunius, M. de Ridder, Eduard H. J. Voormolen, Remco de Bree, Ronald L. A. W. Bleys, Rachel Kalmann, Gerben E. Breimer, Johannes A. Rijken

**Affiliations:** ^1^Department of Head and Neck Surgical Oncology, University Medical Center Utrecht, Utrecht, Netherlands; ^2^Department of Radiation Oncology, University Medical Center Utrecht, Utrecht, Netherlands; ^3^Department of Neurosurgery, University Medical Center Utrecht, Utrecht, Netherlands; ^4^Department of Anatomy, University Medical Center Utrecht, Utrecht, Netherlands; ^5^Department of Ophthalmology, University Medical Center Utrecht, Utrecht, Netherlands; ^6^Department of Pathology, University Medical Center Utrecht, Utrecht, Netherlands

**Keywords:** lymph node metastases, mucosal melanoma, paranasal sinus neoplasm, sinonasal cancer

## Abstract

The following case potentially provides insight into the mechanisms of lymphogenic metastasis in sinonasal cancer. A 63-year-old patient who presented with progressive diplopia and left-sided periocular pain was diagnosed with a cT4bN0M0 mucosal melanoma of the ethmoid sinus. She underwent a combined endonasal and transcranial tumor resection, and an orbital exenteration. Upon histopathological examination, besides the primary tumor, two separate localizations of melanoma surrounded by lymphoid tissues and lymph follicles were identified. The tumor was upstaged to pT4bN1, and the patient received a combination of adjuvant immunotherapy and radiotherapy. At present, the patient displays no evidence of disease. The presence of orbital lymph nodes has previously never been confirmed. These findings indicate the potential involvement of lymphatic drainage through the retrobulbar fat in the regional spread of sinonasal tumors closely associated with the orbit.

## 1. Introduction

Sinonasal malignancies are a rare and heterogeneous group of diseases within the overarching spectrum of head and neck cancer. One of the key differences is the general rarity of lymph node metastases at first presentation. Moreover, the route of lymphogenic metastasis from the nasal cavity and paranasal sinuses remains largely unclear. The following case constitutes a novel finding that may provide more insight into the mechanisms of regional spread in sinonasal cancer.

## 2. Patient Information

A 63-year-old female patient was referred to the outpatient clinic of a tertiary referral hospital. She presented with progressive diplopia and left-sided periocular pain, both of which had been present for 2 months. She also experienced left-sided nose obstruction for several years, but no other symptoms. Besides an appendectomy 20 years prior and mild obstructive sleep apnea, the patient had no other pre-existing somatic, psychiatric, or familial conditions.

### 2.1. Clinical Findings

**Figure 1:**
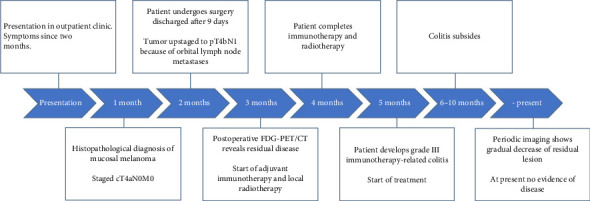
Timeline of patient history and treatment.

Physical examination, including nasolaryngoscopy and ophthalmological assessment, revealed impaired abduction of the left eye with an exacerbation of diplopia, but no other relevant findings. Notably, the patient's vision and other cranial nerve functions were intact. A complete timeline of further treatment is shown in Figure 1.

### 2.2. Diagnostic Assessment

**Figure 2:**
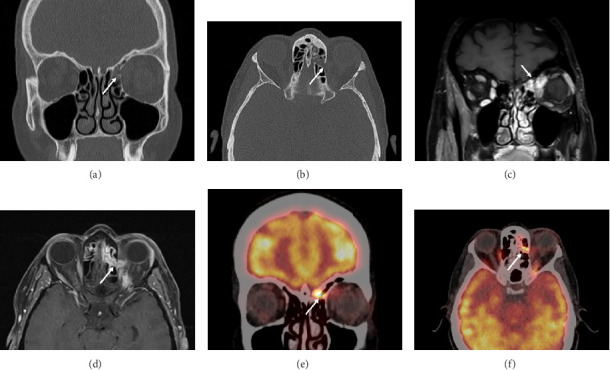
Imaging studies of a patient with a cT4bN0M0 mucosal melanoma of the ethmoid sinus: (a) coronal CT image; (b) axial CT image; (c) coronal postcontrast T1-weighted MR image with fat saturation (T1 MR Gd FS); (d) axial T1 MR Gd FS image; (e) coronal 18F-FDG PET/CT; (f) axial coronal 18F-FDG PET/CT; white arrows point toward the primary tumor.

Magnetic resonance imaging (MRI) and computed tomography (CT) of the head and neck revealed a tumor of the left ethmoid sinus with extension through the lamina papyracea to the orbit (Figure 2). Cranially, the tumor extended to the anterior skull base with invasion of the cribriform plate. A fluor-18-deoxyglucose (FDG) positron emission tomography (PET)-CT showed no signs of regional or distant metastases. Histopathological examination of a biopsy, which had been acquired via an anterior orbitotomy, resulted in a diagnosis of malignant melanoma. The tumor was staged in accordance with the eighth edition of the UICC TNM classification as a cT4bN0M0 sinonasal mucosal melanoma.

### 2.3. Therapeutic Intervention

**Figure 3:**
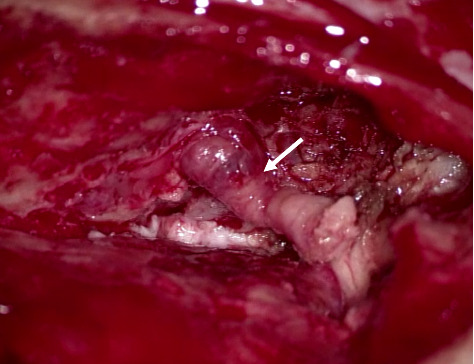
Peroperative view of the sinonasal lesion as seen cranially after craniofacial resection. The white arrow points at the lesion.

The patient underwent a combined endonasal and transcranial tumor resection with anterior skull base reconstruction with a galea flap and titanium mesh, orbital exenteration, and placement of implants for epithesis. The procedure was carried out by a team consisting of a head and neck surgeon, a neurosurgeon, and an orbital surgeon. Macroscopic radical tumor resection was achieved. Peroperatively, the lesion could clearly be identified as it extended towards the left orbit (Figure 3). A split skin graft was placed to cover the remaining orbital defect. Histopathological examination confirmed the diagnosis of mucosal melanoma. Notably, two separate metastases of mucosal melanoma were found within the resected orbital contents. Since these lesions, the largest of which measured 6.6 mm, were surrounded by lymphoid tissue and lymph follicles, they were considered to be orbital lymph node metastases. The tumor was subsequently upstaged to pT4bN1. Postoperative FDG-PET/CT revealed suspected residual disease in the orbital apex with extension along the optic nerve and ophthalmic vein. Following discussion in the multidisciplinary tumor board, the patient received a combination of adjuvant local radiotherapy (3600 cGy in 6 fractions) and concomitant immunotherapy (ipilimumab/nivolumab).

### 2.4. Follow-Up and Outcomes

One month after starting immunotherapy, the patient developed Grade III colitis which subsided following treatment with prednisone, infliximab, and tacrolimus. Periodic MRI and CT during follow-up showed a steady decrease of the residual lesion. At the time of writing, nearly 3 years after initial treatment, the patient remains in follow-up without signs of disease progression.

## 3. Discussion

**Figure 4:**
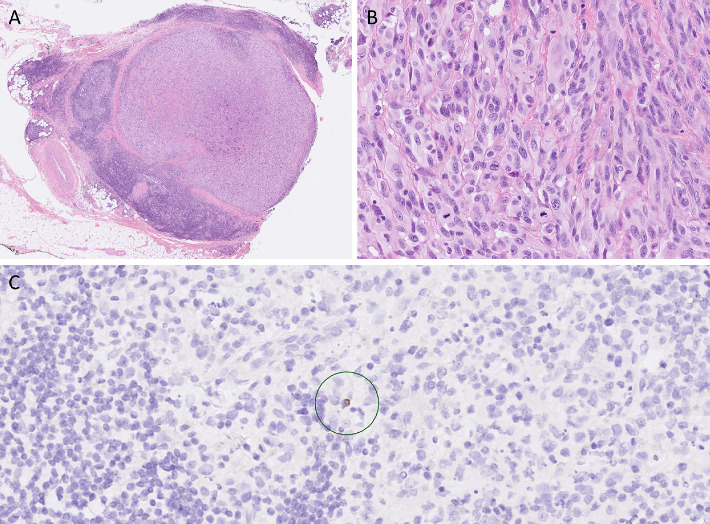
Histopathological images of the orbital metastasis and surrounding tissues: (A) H&E stain of one orbital metastasis of mucosal melanoma surrounded by lymphoid tissue; (B) H&E stain of detail of melanoma tumor cells; (C) cytokeratin 8/18 stain of the lymphoid tissue displaying sporadic presence of keratin-positive cells (circled in green).

Distinguishing between tumor-associated lymphoid tissue and actual lymph nodes can be achieved by examining the presence of keratin-positive extrafollicular reticulum cells. Immunostaining for low molecular weight keratin shows extrafollicular reticulum cells in lymph nodes, whereas this is not the case in reactive lymphoid proliferations [1]. Therefore, additional cytokeratin AE1/3 and cytokeratin 8/18 immunostainings were performed (Figure 4). Because keratin-positive cells were only sporadically encountered in these lesions, their significance remains unclear.

The scientific literature regarding orbital lymphatics is scarce. Except for lymphatic-like structures in the extension of the dura mater surrounding the optic nerve, the orbital fat is seemingly devoid of lymphatic vasculature [2]. Transient expression of lymphatic vessel markers has been observed in the orbital intraconal orbital contents during the fetal and early neonatal period, but their expression was absent in adult tissue samples [3]. Lymphangiogenesis, however, has shown to occur in the orbital fat under the influence of inflammatory conditions or infection, suggesting a certain degree of plasticity [4, 5].

The presence of orbital lymph nodes has never been confirmed, raising the possibility that the observed metastases in this case might be in-transit metastases. The lymph node–like background could be attributed to the formation of the tumor-associated lymphoid tissue, also known as tertiary lymphoid structures, which have been observed in various types of cancer [6]. However, despite conducting additional immunohistochemical stainings, it was not possible to definitively determine whether these structures represent a lymph node or tumor-associated lymphoid tissue. Based on the existing literature, it seems unlikely that they are lymph nodes. Tertiary lymphoid structures have been identified as major players in anti–tumor immune responses. Although the underlying mechanism of their contribution requires further investigation, their presence or induction following (immune) therapy has shown to correlate with favorable outcomes [7].

Initially, the two separate localizations of mucosal melanoma within the orbital fat were considered lymph node metastases and treated accordingly. In hindsight, this assessment may have been incorrect. Regardless, these findings indicate the potential involvement of lymphatic drainage through the retrobulbar fat in the regional spread of sinonasal tumors closely associated with the orbit. This case represents the first reported instance of its kind and offers valuable insights into the mechanisms of regional metastasis in sinonasal cancer.

### 3.1. Patient Perspective

The patient is relieved that treatment up to now has been successful. Although imaging during follow-up shows no signs of recurrent disease, she remains anxious that the disease might return. The patient also struggled with the removal of her eye. The epithesis came out beautifully, and friends and family barely notice, but it is still not her own eye.

## Data Availability

Data will not be made available.
